# Chlorido{*N*-[2-(diphenylphosphanyl)benz­ylidene]-2-(2-thienyl)ethanamine-κ^2^ *N*,*P*}methylpalladium(II) dichloromethane hemisolvate

**DOI:** 10.1107/S1600536810017824

**Published:** 2010-05-22

**Authors:** Martin O. Onani, William M. Motswainyana, Emmanuel I. Iwuoha, James Darkwa, Roger A. Lalancette

**Affiliations:** a University of the Western Cape, Cape Town, Bellville 7535, South Africa; b University of Johannesburg, Auckland Park Kingsway Campus, Johannesburg 2006, South Africa; c Department of Chemistry, Rutgers State University, 73 Warren St, Newark, NJ 07102, USA

## Abstract

In the title compound, [Pd(CH_3_)Cl(C_25_H_22_NPS)]·0.5C_2_H_2_Cl_2_, the Pd^II^ atom is coordinated by the *N*,*P*-bidentate ligand, a methyl group and a chloride ion, generating a distorted square-planar PdCClNS coordination geometry, with the N and Cl atoms *trans*. The thio­phene ring is equally disordered over two orientations and the dichloro­methane solvent mol­ecule is disordered about an inversion centre.

## Related literature

For metal-organic compounds with ligands containing both pyridyl and phosphine donor groups and for typical Pd—C, Pd—Cl, Pd—P and Pd—N bond lengths, see: Shaffer & Schmidt (2009 ▶). For the properties of related compounds, see: Tongwa *et al.* (2009 ▶); Jun-Gill *et al.* (2009 ▶).
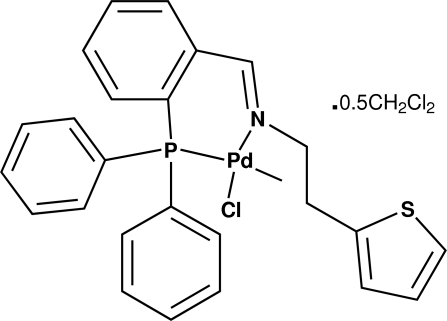


## Experimental

### 

#### Crystal data

[Pd(CH_3_)Cl(C_25_H_22_NPS)]·0.5C_2_H_2_Cl_2_
*M* *_r_* = 598.81
Monoclinic, 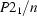

*a* = 9.9960 (6) Å
*b* = 18.6584 (11) Å
*c* = 13.8167 (8) Å
β = 94.517 (1)°
*V* = 2568.9 (3) Å^3^
*Z* = 4
Mo *K*α radiation
μ = 1.09 mm^−1^
*T* = 173 K
0.16 × 0.15 × 0.14 mm

#### Data collection

Bruker Kappa DUO APEXII CCD diffractometer
Absorption correction: multi-scan (*SADABS*; Bruker, 2006 ▶) *T*_min_ = 0.683, *T*_max_ = 0.746
31867 measured reflections
6376 independent reflections
5600 reflections with *I* > 2σ(*I*)
*R*_int_ = 0.028

#### Refinement

*R*[*F* ^2^ > 2σ(*F* ^2^)] = 0.029
*wR*(*F* ^2^) = 0.074
*S* = 1.03
6376 reflections
321 parameters
2 restraints
H-atom parameters constrained
Δρ_max_ = 1.34 e Å^−3^
Δρ_min_ = −1.04 e Å^−3^

### 

Data collection: *SMART* (Bruker, 2006 ▶); cell refinement: *SAINT* (Bruker, 2006 ▶); data reduction: *SAINT*; program(s) used to solve structure: *SHELXS97* (Sheldrick, 2008 ▶); program(s) used to refine structure: *SHELXL97* (Sheldrick, 2008 ▶); molecular graphics: *X-SEED* (Barbour, 2001 ▶); software used to prepare material for publication: *SHELXL97*.

## Supplementary Material

Crystal structure: contains datablocks I, global. DOI: 10.1107/S1600536810017824/hb5421sup1.cif

Structure factors: contains datablocks I. DOI: 10.1107/S1600536810017824/hb5421Isup2.hkl

Additional supplementary materials:  crystallographic information; 3D view; checkCIF report

## Figures and Tables

**Table 1:** Selected bond lengths (Å)

Pd1—C1	2.045 (2)
Pd1—N1	2.158 (2)
Pd1—P1	2.2039 (6)
Pd1—Cl1	2.3628 (6)

**Fig. 1.**
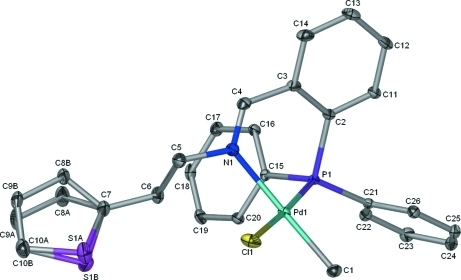
: The molecular structure of (I) showing 30% displacement ellipsoids (all hydrogen atoms omitted for clarity). The solvent dichloromethane molecule is excluded.

## supplementary crystallographic information

### Comment

The stucture of the title compound, (I), is shown below. Dimensions are available in the archived CIF. The solvent molecule dichloromethane exhibits high thermal motions and were refined isotropically with temperature factors in the range of 0.101 – 0.122. It is situated close to a centre of inversion. The five-membered ring was disordered and shows two orientations each at 50% s.o.f.: the first ring C7, C8A, C9A, C10A and S1A (ring A) and the second ring C7, C8B, C9B, C10B and S1B (ring B). Ring A and ring B share two common atom sites at C7 and C10A (or C10B). C10A and C10B are on the same site and refined anisotropically with the same temperature factors. The maximum and minimum deviations from the least-squares planes of both rings are 0.086 (4) Å and -0.084 (3) Å for C7 and S1A in ring A, 0.095 (5) Å and -0.090 (3) Å for C7 and S1B in ring B. Angle from the least-square plane of ring A to that of ring B is 36.2 (3)°.

### Experimental

The iminophosphine heterocyclic ligand was prepared via the condensation reaction of 2-(diphenylphosphino)benzaldehyde with 2-thien-2-ylethanamine. The ligand was further refluxed with an equimolar Pd(cod)Cl_2_ in dichloromethane and gave over 80% yield of a yellow complex. Light-yellow blocks of (I) were grown via slow diffusion of a dichloromethane solution of the complex in hexane a 4

### Refinement

The solvent molecule dichloromethane exhibits high thermal motions and were refined isotropically with temperature factors in the range of 0.101 – 0.122. It is situated on the centre of inversion. Therefore only half of the molecule is in the asymmetric unit and it is modelled as a whole molecule with 50% site occupancy factor (s.o.f.). The 5 member ring was disordered and shows two preferred orientations each at 50% s.o.f.: the first ring C7, C8A, C9A, C10A and S1A (ring A) and the second ring C7, C8B, C9B, C10B and S1B (ring B). Ring A and ring B share two common atom sites at C7 and C10A (or C10B). C10A and C10B are on the same site and refined anisotropically with the same temperature factors. The maximum and minimum deviations from the least-squares planes of both rings are 0.086 (4) Å and -0.084 (3) Å for C7 and S1A in ring A, 0.095 (5) Å and -0.090 (3) Å for C7 and S1B in ring B. Angle from the least-square plane of ring A to that of ring B is 36.2 (3)o. All hydrogen atoms were positioned geometrically with C—H = 0.95 – 0.99 Å and refined as riding on their parent atoms with Uiso (H) = 1.2 - 1.5 Ueq (C).

### Crystal data

**Table:**

[Pd(CH_3_)Cl(C_25_H_22_NPS)]·0.5C_2_H_2_Cl_2_	*F*(000) = 1212
*M**_r_* = 598.81	*D*_x_ = 1.548 Mg m^−^^3^
Monoclinic, *P*2_1_/*n*	Mo *K*α radiation, λ = 0.71073 Å
Hall symbol: -P 2yn	Cell parameters from 31867 reflections
*a* = 9.9960 (6) Å	θ = 2.2–28.3°
*b* = 18.6584 (11) Å	µ = 1.09 mm^−^^1^
*c* = 13.8167 (8) Å	*T* = 173 K
β = 94.517 (1)°	Needle, light-yellow
*V* = 2568.9 (3) Å^3^	0.16 × 0.15 × 0.14 mm
*Z* = 4	

### Data collection

**Table:**

Bruker Kappa DUO APEXII CCD diffractometer	6376 independent reflections
Radiation source: fine-focus sealed tube	5600 reflections with *I* > 2σ(*I*)
graphite	*R*_int_ = 0.028
0.5° φ scans and ω scans	θ_max_ = 28.3°, θ_min_ = 2.2°
Absorption correction: multi-scan (*SADABS*; Bruker, 2006)	*h* = −13→13
*T*_min_ = 0.683, *T*_max_ = 0.746	*k* = −24→24
31867 measured reflections	*l* = −18→18

### Refinement

**Table:**

Refinement on *F*^2^	Primary atom site location: structure-invariant direct methods
Least-squares matrix: full	Secondary atom site location: difference Fourier map
*R*[*F*^2^ > 2σ(*F*^2^)] = 0.029	Hydrogen site location: inferred from neighbouring sites
*wR*(*F*^2^) = 0.074	H-atom parameters constrained
*S* = 1.03	*w* = 1/[σ^2^(*F*_o_^2^) + (0.0301*P*)^2^ + 3.2924*P*] where *P* = (*F*_o_^2^ + 2*F*_c_^2^)/3
6376 reflections	(Δ/σ)_max_ = 0.002
321 parameters	Δρ_max_ = 1.34 e Å^−^^3^
2 restraints	Δρ_min_ = −1.04 e Å^−^^3^

### Special details

**Table:**

Experimental. Half sphere of data collected using SAINT strategy (Bruker, 2006). Crystal to detector distance = 50 mm; combination of φ and ω scans of 0.5°, 50 s per °, 2 iterations.
Geometry. All esds (except the esd in the dihedral angle between two l.s. planes) are estimated using the full covariance matrix. The cell esds are taken into account individually in the estimation of esds in distances, angles and torsion angles; correlations between esds in cell parameters are only used when they are defined by crystal symmetry. An approximate (isotropic) treatment of cell esds is used for estimating esds involving l.s. planes.
Refinement. The solvent molecule dichloromethane exhibits high thermal motions and were refined isotropically with temperature factors in the range of 0.101 C 0.122. It is modelled as a whole molecule with 50% s.o.f. The 5 member ring was disordered and shows two preferred orientations each at 50% s.o.f. All hydrogen atoms were positioned geometrically with C—H = 0.95 C 0.99 A and refined as riding on their parent atoms with Uiso (H) = 1.2 - 1.5 Ueq (C). Refinement of *F*^2^ against ALL reflections. The weighted *R*-factor wR and goodness of fit *S* are based on *F*^2^, conventional *R*-factors *R* are based on *F*, with *F* set to zero for negative *F*^2^. The threshold expression of *F*^2^ > σ(*F*^2^) is used only for calculating *R*-factors(gt) etc. and is not relevant to the choice of reflections for refinement. *R*-factors based on *F*^2^ are statistically about twice as large as those based on *F*, and *R*- factors based on ALL data will be even larger.

### Fractional atomic coordinates and isotropic or equivalent isotropic displacement parameters (Å^2^)

**Table:**

	*x*	*y*	*z*	*U*_iso_*/*U*_eq_	Occ. (<1)
Pd1	0.636610 (16)	0.296906 (8)	0.583217 (12)	0.02079 (5)	
Cl1	0.51346 (6)	0.20048 (3)	0.64253 (6)	0.03899 (15)	
Cl2A	1.0260 (4)	0.55967 (18)	1.0814 (2)	0.1066 (9)*	0.50
Cl2B	0.9080 (4)	0.4537 (2)	0.9486 (3)	0.1223 (11)*	0.50
S1A	0.3721 (8)	0.3256 (4)	0.9098 (5)	0.0464 (12)	0.50
S1B	0.3499 (8)	0.3404 (4)	0.8969 (6)	0.0443 (12)	0.50
P1	0.76594 (5)	0.38331 (3)	0.53464 (4)	0.01817 (11)	
N1	0.7551 (2)	0.30566 (11)	0.72019 (14)	0.0289 (4)	
C1	0.5273 (2)	0.28695 (14)	0.45247 (18)	0.0316 (5)	
H1A	0.5795	0.2602	0.4074	0.047*	
H1B	0.5062	0.3347	0.4259	0.047*	
H1C	0.4438	0.2612	0.4615	0.047*	
C2	0.9364 (2)	0.34988 (11)	0.56117 (16)	0.0214 (4)	
C3	0.9725 (2)	0.31802 (13)	0.65212 (17)	0.0268 (5)	
C4	0.8828 (3)	0.31090 (14)	0.73071 (18)	0.0315 (5)	
H4	0.9235	0.3101	0.7952	0.038*	
C5	0.6819 (3)	0.30364 (16)	0.80910 (18)	0.0374 (6)	
H5A	0.7465	0.3037	0.8672	0.045*	
H5B	0.6275	0.2594	0.8100	0.045*	
C6	0.5910 (3)	0.36912 (17)	0.81044 (19)	0.0402 (6)	
H6A	0.6467	0.4130	0.8089	0.048*	
H6B	0.5284	0.3689	0.7513	0.048*	
C7	0.5115 (3)	0.37153 (19)	0.8982 (2)	0.0455 (7)	
C8A	0.5216 (8)	0.4289 (5)	0.9701 (5)	0.0549 (18)	0.50
H8A	0.5839	0.4674	0.9708	0.066*	0.50
C8B	0.5611 (7)	0.3773 (5)	0.9925 (5)	0.059 (2)	0.50
H8B	0.6537	0.3845	1.0113	0.070*	0.50
C9A	0.4239 (8)	0.4189 (5)	1.0396 (5)	0.0569 (19)	0.50
H9A	0.4164	0.4492	1.0942	0.068*	0.50
C9B	0.4608 (7)	0.3717 (5)	1.0608 (4)	0.0522 (17)	0.50
H9B	0.4791	0.3767	1.1290	0.063*	0.50
C10A	0.3397 (3)	0.35877 (19)	1.0181 (2)	0.0497 (8)	0.50
H10A	0.2753	0.3403	1.0586	0.060*	0.50
C10B	0.3397 (3)	0.35877 (19)	1.0181 (2)	0.0497 (8)	0.50
H10B	0.2589	0.3595	1.0499	0.060*	0.50
C11	1.0318 (2)	0.35515 (12)	0.49348 (17)	0.0263 (5)	
H11	1.0084	0.3770	0.4324	0.032*	
C12	1.1615 (2)	0.32884 (14)	0.5141 (2)	0.0323 (5)	
H12	1.2250	0.3320	0.4666	0.039*	
C13	1.1980 (3)	0.29822 (14)	0.6033 (2)	0.0380 (6)	
H13	1.2863	0.2803	0.6175	0.046*	
C14	1.1042 (3)	0.29394 (14)	0.6720 (2)	0.0358 (6)	
H14	1.1301	0.2742	0.7340	0.043*	
C15	0.7553 (2)	0.46247 (11)	0.61019 (15)	0.0195 (4)	
C16	0.8684 (2)	0.49563 (12)	0.65553 (16)	0.0250 (4)	
H16	0.9552	0.4770	0.6473	0.030*	
C17	0.8542 (3)	0.55604 (13)	0.71283 (17)	0.0306 (5)	
H17	0.9313	0.5782	0.7444	0.037*	
C18	0.7276 (3)	0.58398 (13)	0.72402 (17)	0.0309 (5)	
H18	0.7185	0.6258	0.7621	0.037*	
C19	0.6147 (3)	0.55098 (13)	0.67984 (17)	0.0300 (5)	
H19	0.5281	0.5699	0.6883	0.036*	
C20	0.6280 (2)	0.49029 (12)	0.62321 (16)	0.0249 (4)	
H20	0.5504	0.4676	0.5932	0.030*	
C21	0.7591 (2)	0.41830 (11)	0.41116 (15)	0.0203 (4)	
C22	0.7452 (2)	0.49174 (12)	0.39294 (16)	0.0243 (4)	
H22	0.7424	0.5244	0.4455	0.029*	
C23	0.7355 (3)	0.51719 (13)	0.29770 (17)	0.0312 (5)	
H23	0.7263	0.5671	0.2856	0.037*	
C24	0.7393 (3)	0.46996 (15)	0.22089 (17)	0.0328 (5)	
H24	0.7310	0.4874	0.1561	0.039*	
C25	0.7550 (3)	0.39725 (15)	0.23840 (17)	0.0320 (5)	
H25	0.7593	0.3650	0.1855	0.038*	
C26	0.7644 (2)	0.37123 (13)	0.33299 (17)	0.0268 (5)	
H26	0.7744	0.3212	0.3445	0.032*	
C27	0.9815 (11)	0.5338 (5)	0.9658 (6)	0.101 (3)*	0.50
H27A	0.9199	0.5705	0.9358	0.121*	0.50
H27B	1.0632	0.5342	0.9297	0.121*	0.50

### Atomic displacement parameters (Å^2^)

**Table:**

	*U*^11^	*U*^22^	*U*^33^	*U*^12^	*U*^13^	*U*^23^
Pd1	0.01711 (8)	0.01957 (8)	0.02575 (9)	0.00040 (6)	0.00207 (6)	0.00374 (6)
Cl1	0.0251 (3)	0.0310 (3)	0.0611 (4)	−0.0026 (2)	0.0048 (3)	0.0200 (3)
S1A	0.058 (3)	0.0360 (18)	0.048 (3)	−0.0159 (16)	0.022 (2)	−0.0098 (16)
S1B	0.0410 (17)	0.061 (4)	0.0305 (10)	−0.0139 (19)	0.0016 (10)	0.0018 (19)
P1	0.0166 (2)	0.0187 (2)	0.0192 (2)	0.00054 (19)	0.00097 (18)	0.00085 (19)
N1	0.0272 (10)	0.0343 (11)	0.0252 (10)	0.0028 (8)	0.0030 (8)	0.0106 (8)
C1	0.0256 (11)	0.0375 (13)	0.0310 (12)	−0.0099 (10)	−0.0026 (9)	−0.0016 (10)
C2	0.0186 (9)	0.0199 (9)	0.0257 (10)	0.0007 (8)	0.0004 (8)	−0.0008 (8)
C3	0.0216 (11)	0.0281 (11)	0.0303 (12)	0.0032 (9)	−0.0013 (9)	0.0041 (9)
C4	0.0290 (12)	0.0395 (13)	0.0254 (11)	0.0054 (10)	−0.0024 (9)	0.0104 (10)
C5	0.0343 (13)	0.0532 (16)	0.0253 (12)	0.0016 (12)	0.0052 (10)	0.0140 (11)
C6	0.0427 (15)	0.0537 (17)	0.0254 (12)	0.0045 (13)	0.0099 (11)	0.0064 (11)
C7	0.0359 (14)	0.074 (2)	0.0269 (13)	−0.0073 (14)	0.0076 (11)	−0.0028 (13)
C8A	0.054 (4)	0.072 (5)	0.042 (4)	−0.028 (4)	0.018 (3)	−0.014 (3)
C8B	0.032 (3)	0.114 (7)	0.031 (3)	−0.017 (4)	0.010 (2)	−0.018 (4)
C9A	0.065 (5)	0.075 (5)	0.034 (3)	−0.019 (4)	0.022 (3)	−0.016 (3)
C9B	0.045 (4)	0.089 (6)	0.024 (3)	−0.014 (4)	0.010 (2)	−0.010 (3)
C10A	0.0469 (17)	0.067 (2)	0.0378 (15)	−0.0110 (15)	0.0179 (13)	−0.0025 (14)
C10B	0.0469 (17)	0.067 (2)	0.0378 (15)	−0.0110 (15)	0.0179 (13)	−0.0025 (14)
C11	0.0225 (11)	0.0270 (11)	0.0295 (11)	0.0006 (9)	0.0025 (9)	−0.0015 (9)
C12	0.0209 (11)	0.0333 (12)	0.0434 (14)	0.0029 (9)	0.0072 (10)	−0.0065 (11)
C13	0.0214 (11)	0.0363 (13)	0.0559 (17)	0.0080 (10)	−0.0001 (11)	0.0021 (12)
C14	0.0263 (12)	0.0386 (14)	0.0413 (14)	0.0075 (10)	−0.0057 (10)	0.0095 (11)
C15	0.0212 (10)	0.0198 (9)	0.0178 (9)	−0.0001 (8)	0.0030 (7)	0.0008 (7)
C16	0.0220 (10)	0.0264 (11)	0.0265 (11)	−0.0005 (8)	0.0025 (8)	−0.0032 (9)
C17	0.0325 (12)	0.0308 (12)	0.0285 (12)	−0.0069 (10)	0.0022 (9)	−0.0070 (9)
C18	0.0439 (14)	0.0256 (11)	0.0246 (11)	−0.0011 (10)	0.0107 (10)	−0.0051 (9)
C19	0.0307 (12)	0.0294 (11)	0.0310 (12)	0.0074 (10)	0.0097 (10)	−0.0001 (9)
C20	0.0211 (10)	0.0277 (11)	0.0261 (11)	0.0021 (8)	0.0029 (8)	0.0011 (9)
C21	0.0165 (9)	0.0240 (10)	0.0204 (10)	0.0002 (8)	0.0012 (7)	0.0012 (8)
C22	0.0262 (11)	0.0237 (10)	0.0228 (10)	−0.0029 (8)	0.0009 (8)	0.0007 (8)
C23	0.0344 (13)	0.0294 (12)	0.0295 (12)	−0.0015 (10)	0.0008 (10)	0.0079 (9)
C24	0.0313 (12)	0.0450 (14)	0.0224 (11)	0.0006 (11)	0.0041 (9)	0.0051 (10)
C25	0.0310 (12)	0.0434 (14)	0.0219 (11)	0.0052 (11)	0.0032 (9)	−0.0050 (10)
C26	0.0258 (11)	0.0279 (11)	0.0267 (11)	0.0052 (9)	0.0027 (9)	−0.0022 (9)

### Geometric parameters (Å, °)

**Table:**

Pd1—C1	2.045 (2)	C9A—H9A	0.9500
Pd1—N1	2.158 (2)	C9B—H9B	0.9500
Pd1—P1	2.2039 (6)	C10A—H10A	0.9500
Pd1—Cl1	2.3628 (6)	C11—C12	1.394 (3)
Cl2A—C27	1.695 (7)	C11—H11	0.9500
Cl2B—C27	1.673 (8)	C12—C13	1.381 (4)
S1A—C7	1.655 (9)	C12—H12	0.9500
S1A—C10A	1.673 (9)	C13—C14	1.388 (4)
S1B—C7	1.715 (9)	C13—H13	0.9500
P1—C15	1.817 (2)	C14—H14	0.9500
P1—C21	1.823 (2)	C15—C16	1.394 (3)
P1—C2	1.825 (2)	C15—C20	1.398 (3)
N1—C4	1.278 (3)	C16—C17	1.391 (3)
N1—C5	1.479 (3)	C16—H16	0.9500
C1—H1A	0.9800	C17—C18	1.388 (4)
C1—H1B	0.9800	C17—H17	0.9500
C1—H1C	0.9800	C18—C19	1.384 (4)
C2—C11	1.391 (3)	C18—H18	0.9500
C2—C3	1.411 (3)	C19—C20	1.389 (3)
C3—C14	1.397 (3)	C19—H19	0.9500
C3—C4	1.467 (3)	C20—H20	0.9500
C4—H4	0.9500	C21—C26	1.396 (3)
C5—C6	1.523 (4)	C21—C22	1.398 (3)
C5—H5A	0.9900	C22—C23	1.395 (3)
C5—H5B	0.9900	C22—H22	0.9500
C6—C7	1.502 (4)	C23—C24	1.382 (4)
C6—H6A	0.9900	C23—H23	0.9500
C6—H6B	0.9900	C24—C25	1.385 (4)
C7—C8B	1.362 (7)	C24—H24	0.9500
C7—C8A	1.459 (8)	C25—C26	1.390 (3)
C8A—C9A	1.434 (9)	C25—H25	0.9500
C8A—H8A	0.9500	C26—H26	0.9500
C8B—C9B	1.434 (8)	C27—H27A	0.9900
C8B—H8B	0.9500	C27—H27B	0.9900
C9A—C10A	1.420 (8)		
			
C1—Pd1—N1	178.73 (9)	C10A—C9A—H9A	123.7
C1—Pd1—P1	94.76 (7)	C8A—C9A—H9A	123.7
N1—Pd1—P1	85.25 (6)	C8B—C9B—H9B	123.8
C1—Pd1—Cl1	88.88 (7)	C9A—C10A—S1A	109.1 (4)
N1—Pd1—Cl1	91.05 (6)	C9A—C10A—H10A	125.5
P1—Pd1—Cl1	175.36 (2)	S1A—C10A—H10A	125.5
C7—S1A—C10A	96.8 (4)	C2—C11—C12	120.9 (2)
C15—P1—C21	104.30 (10)	C2—C11—H11	119.6
C15—P1—C2	104.99 (10)	C12—C11—H11	119.6
C21—P1—C2	106.03 (10)	C13—C12—C11	120.3 (2)
C15—P1—Pd1	110.94 (7)	C13—C12—H12	119.9
C21—P1—Pd1	124.56 (7)	C11—C12—H12	119.9
C2—P1—Pd1	104.47 (7)	C12—C13—C14	119.3 (2)
C4—N1—C5	117.6 (2)	C12—C13—H13	120.4
C4—N1—Pd1	125.45 (17)	C14—C13—H13	120.4
C5—N1—Pd1	116.96 (16)	C13—C14—C3	121.6 (2)
Pd1—C1—H1A	109.5	C13—C14—H14	119.2
Pd1—C1—H1B	109.5	C3—C14—H14	119.2
H1A—C1—H1B	109.5	C16—C15—C20	119.4 (2)
Pd1—C1—H1C	109.5	C16—C15—P1	122.46 (16)
H1A—C1—H1C	109.5	C20—C15—P1	118.09 (16)
H1B—C1—H1C	109.5	C17—C16—C15	120.0 (2)
C11—C2—C3	119.2 (2)	C17—C16—H16	120.0
C11—C2—P1	121.32 (17)	C15—C16—H16	120.0
C3—C2—P1	119.46 (17)	C18—C17—C16	120.2 (2)
C14—C3—C2	118.7 (2)	C18—C17—H17	119.9
C14—C3—C4	116.5 (2)	C16—C17—H17	119.9
C2—C3—C4	124.7 (2)	C19—C18—C17	120.2 (2)
N1—C4—C3	125.9 (2)	C19—C18—H18	119.9
N1—C4—H4	117.1	C17—C18—H18	119.9
C3—C4—H4	117.1	C18—C19—C20	120.0 (2)
N1—C5—C6	108.9 (2)	C18—C19—H19	120.0
N1—C5—H5A	109.9	C20—C19—H19	120.0
C6—C5—H5A	109.9	C19—C20—C15	120.2 (2)
N1—C5—H5B	109.9	C19—C20—H20	119.9
C6—C5—H5B	109.9	C15—C20—H20	119.9
H5A—C5—H5B	108.3	C26—C21—C22	119.1 (2)
C7—C6—C5	112.8 (2)	C26—C21—P1	119.85 (17)
C7—C6—H6A	109.0	C22—C21—P1	121.03 (16)
C5—C6—H6A	109.0	C23—C22—C21	120.1 (2)
C7—C6—H6B	109.0	C23—C22—H22	119.9
C5—C6—H6B	109.0	C21—C22—H22	119.9
H6A—C6—H6B	107.8	C24—C23—C22	120.2 (2)
C8B—C7—C8A	44.7 (4)	C24—C23—H23	119.9
C8B—C7—C6	126.8 (4)	C22—C23—H23	119.9
C8A—C7—C6	124.0 (4)	C23—C24—C25	120.0 (2)
C8B—C7—S1A	101.2 (4)	C23—C24—H24	120.0
C8A—C7—S1A	109.1 (4)	C25—C24—H24	120.0
C6—C7—S1A	124.5 (4)	C24—C25—C26	120.3 (2)
C8B—C7—S1B	108.0 (4)	C24—C25—H25	119.8
C8A—C7—S1B	105.6 (4)	C26—C25—H25	119.8
C6—C7—S1B	122.7 (4)	C25—C26—C21	120.2 (2)
S1A—C7—S1B	13.1 (3)	C25—C26—H26	119.9
C9A—C8A—C7	110.3 (6)	C21—C26—H26	119.9
C9A—C8A—H8A	124.8	Cl2B—C27—Cl2A	117.9 (6)
C7—C8A—H8A	124.8	Cl2B—C27—H27A	107.8
C7—C8B—C9B	113.8 (5)	Cl2A—C27—H27A	107.8
C7—C8B—H8B	123.1	Cl2B—C27—H27B	107.8
C9B—C8B—H8B	123.1	Cl2A—C27—H27B	107.8
C10A—C9A—C8A	112.7 (6)	H27A—C27—H27B	107.2
			
C1—Pd1—P1—C15	122.52 (11)	S1B—C7—C8A—C9A	24.7 (8)
N1—Pd1—P1—C15	−58.75 (9)	C8A—C7—C8B—C9B	81.6 (9)
Cl1—Pd1—P1—C15	−96.0 (3)	C6—C7—C8B—C9B	−174.7 (6)
C1—Pd1—P1—C21	−3.25 (11)	S1A—C7—C8B—C9B	−24.3 (9)
N1—Pd1—P1—C21	175.47 (10)	S1B—C7—C8B—C9B	−12.7 (10)
Cl1—Pd1—P1—C21	138.3 (3)	C7—C8A—C9A—C10A	−2.9 (10)
C1—Pd1—P1—C2	−124.85 (11)	C8A—C9A—C10A—S1A	−6.9 (9)
N1—Pd1—P1—C2	53.87 (9)	C7—S1A—C10A—C9A	12.1 (6)
Cl1—Pd1—P1—C2	16.7 (3)	C3—C2—C11—C12	0.6 (3)
C1—Pd1—N1—C4	46 (4)	P1—C2—C11—C12	−179.39 (18)
P1—Pd1—N1—C4	−44.6 (2)	C2—C11—C12—C13	−1.2 (4)
Cl1—Pd1—N1—C4	132.6 (2)	C11—C12—C13—C14	0.0 (4)
C1—Pd1—N1—C5	−133 (4)	C12—C13—C14—C3	1.9 (4)
P1—Pd1—N1—C5	136.99 (18)	C2—C3—C14—C13	−2.5 (4)
Cl1—Pd1—N1—C5	−45.81 (17)	C4—C3—C14—C13	−179.8 (2)
C15—P1—C2—C11	−109.01 (19)	C21—P1—C15—C16	−96.92 (19)
C21—P1—C2—C11	1.0 (2)	C2—P1—C15—C16	14.4 (2)
Pd1—P1—C2—C11	134.17 (17)	Pd1—P1—C15—C16	126.67 (17)
C15—P1—C2—C3	70.99 (19)	C21—P1—C15—C20	83.53 (18)
C21—P1—C2—C3	−178.95 (18)	C2—P1—C15—C20	−165.18 (17)
Pd1—P1—C2—C3	−45.83 (19)	Pd1—P1—C15—C20	−52.88 (18)
C11—C2—C3—C14	1.2 (3)	C20—C15—C16—C17	−0.3 (3)
P1—C2—C3—C14	−178.79 (19)	P1—C15—C16—C17	−179.81 (18)
C11—C2—C3—C4	178.3 (2)	C15—C16—C17—C18	−0.8 (4)
P1—C2—C3—C4	−1.7 (3)	C16—C17—C18—C19	1.3 (4)
C5—N1—C4—C3	−176.1 (2)	C17—C18—C19—C20	−0.8 (4)
Pd1—N1—C4—C3	5.4 (4)	C18—C19—C20—C15	−0.3 (4)
C14—C3—C4—N1	−154.4 (3)	C16—C15—C20—C19	0.8 (3)
C2—C3—C4—N1	28.5 (4)	P1—C15—C20—C19	−179.61 (17)
C4—N1—C5—C6	116.9 (3)	C15—P1—C21—C26	−179.72 (17)
Pd1—N1—C5—C6	−64.6 (3)	C2—P1—C21—C26	69.74 (19)
N1—C5—C6—C7	179.6 (2)	Pd1—P1—C21—C26	−51.2 (2)
C5—C6—C7—C8B	63.5 (7)	C15—P1—C21—C22	−1.3 (2)
C5—C6—C7—C8A	119.0 (5)	C2—P1—C21—C22	−111.86 (18)
C5—C6—C7—S1A	−80.4 (4)	Pd1—P1—C21—C22	127.25 (16)
C5—C6—C7—S1B	−96.0 (4)	C26—C21—C22—C23	0.6 (3)
C10A—S1A—C7—C8B	32.0 (5)	P1—C21—C22—C23	−177.78 (18)
C10A—S1A—C7—C8A	−13.7 (5)	C21—C22—C23—C24	0.1 (4)
C10A—S1A—C7—C6	−176.8 (3)	C22—C23—C24—C25	−1.1 (4)
C10A—S1A—C7—S1B	−90 (3)	C23—C24—C25—C26	1.3 (4)
C8B—C7—C8A—C9A	−75.3 (8)	C24—C25—C26—C21	−0.5 (4)
C6—C7—C8A—C9A	174.6 (6)	C22—C21—C26—C25	−0.5 (3)
S1A—C7—C8A—C9A	11.4 (8)	P1—C21—C26—C25	177.97 (18)
